# Systematic pan-cancer analysis identifies SLC35C1 as an immunological and prognostic biomarker

**DOI:** 10.1038/s41598-023-32375-0

**Published:** 2023-04-01

**Authors:** Mingchen Xie, Fuxu Wang, Bing Chen, Zeyu Wu, Ci Chen, Jian Xu

**Affiliations:** 1 Department of Neurosurgery, The Affiliated Hospital of Qingdao University, Jiangsu Road No. 16, Qingdao, 266003 Shandong Province China; 2 Department of Stomatology, The Affiliated Hospital of Qingdao University, Jiangsu Road No. 16, Qingdao, 266003 Shandong Province China

**Keywords:** CNS cancer, Tumour biomarkers, Immunosurveillance

## Abstract

GDP-amylose transporter protein 1 (SLC35C1) plays an important role in many types of cancer. Therefore, it is clinically important to further investigate the expression profile of SLC35C1 in human tumors to provide new molecular clues for the pathogenesis of glioma. In this study, we performed a comprehensive pan-cancer analysis of SLC35C1 using a series of bioinformatics approaches and validated its differential tissue expression and biological function. The results showed that SLC35C1 was aberrantly expressed in different types of tumors and significantly correlated with overall survival (OS) and progression-free interval (PFI). More importantly, the expression level of SLC35C1 was closely correlated with Tumor Microenvironment (TME), immune infiltration and immune-related genes. In addition, we found that SLC35C1 expression was also closely related to Tumor Mutation Burden (TMB), Microsatellite Instability (MSI) and antitumor drug sensitivity in various cancer types. Functional bioinformatics analysis indicated that SLC35C1 may be involved in multiple signaling pathways and biological processes in glioma. Based on SLC35C1 expression, a risk factor model was found to predict OS of glioma. In addition, in vitro experiments showed that SLC35C1 knockdown significantly inhibited the proliferation, migration and invasive ability of glioma cells, while SLC35C1 overexpression promoted proliferation, migration, invasion and colony formation of glioma cells. Finally, quantitative real-time PCR confirmed that SLC35C1 was highly expressed in gliomas.

## Introduction

Cancer is the primary matter of death and major obstacle influencing the quality of life in every country, and to date, there are no complete cures for cancer^1–3^. In past times, cancer immunotherapy has become the leading matter, whose security and efficacy have been gradually recognized^4^. With the considerable usage of genome sequencing technology, it is possible to identify new immunotherapy biomarkers through performing pan-cancer expression study of genes and evaluating their correlations with clinical significance and related signaling pathways.

SLC35C1, also admitted as GDP-fucose transporter protein 1, CDG 2C or FUCT 1, is a component of the solute carrier (SLC) histones. It was first cloned from patients with leukocyte adhesion deficiency type II (LAD II) who exhibited reduced GDP-fucose transport to the Golgi apparatus. Immunofluorescence revealed that SLC35C1 is primarily localized to secretion-related subcellular structures, including the Golgi apparatus, endoplasmic reticulum, and endosomes. Some recent studies have shown that SLC35C1 is found to be a negative driver of the classical Wnt pathway in colon cancer species and that deletion of SLC35C1 promotes colon cancer progression by activating the Wnt signaling pathway^5^. Other evidence suggests that SLC35C1 is upexpressed in certain types of cancer and that its high expression is associated with metastasis, poor prognosis and resistance to therapy^6^.

The tumor microenvironment (TME) is known as the “ecological niche” surrounding the tumor, which includes multiple cell types, supportive matrix, as well as soluble factors^7^. TME contains a complex immune cell microenvironment, such as innate immune response cells, such as natural killer (NK) cells and dendritic cells; these cells also play important roles in adaptive immune responses, such as CD8+ and CD4+ T cell^8^. Studies have exhibited that WASF2 is associated with the tumor immune microenvironment. Knockdown of SLC35C1 in immune cells can accelerate severe autoimmunity. Therefore, SLC35C1 may be related to the regulation of tumor immune microenvironment.

Additionally, most analysis studies into the role of SLC35C1 in tumors to date have been limited to a specific kind of tumor. There has been no pan-cancer research of the association between SLC35C1 and various tumors. Thus, multi-omics pan-cancer analysis SLC35C1 not only helps to identify common phenotypic features of tumors, but also provides insight into the genesis of important molecular events and their own regulatory mechanisms. In this analysis, we comprehensively analyzed the expression levels of SLC35C1 in different kinds of cancers and its relationship with prognosis in several datasets such as TCGA, Genotype Tissue Expression (GTEx), and Cancer Cell Line Encyclopedia (CCLE). We also identified the relationship between gene expression and the level of immune infiltration, drug sensitivity and tumor mutational load (TMB) in 33 cancers. Genomic enrichment analysis (GSEA) was also used to explore underlying mechanisms. Finally, we made a prediction model based on SLC35C1 expression and clinical symptoms to predict the prognosis of glioma, and further constructed its regulatory mechanism in glioma progression, and confirmed that SLC35C1 was highly expressed in glioma. In addition, we found that SLC35C1 gene silencing and gene overexpression had opposite effects on the biological behaviour of glioma cells.

## Manuscript formatting

### Materials and methods

#### Sample information and SLC35C1 expression study in human pan-cancer

The TCGA database (https://portal.gdc.cancer.gov/) is presently the largest database of cancer gene information, storing data such as gene expression data, copy number variation, and SNP. We downloaded the original mRNA data and SNP data of 33 types of tumor data in pan-cancer for subsequent research. The gene expression data of each tissue was inquired from the GTEX database (https://commonfund.nih.gov/GTEx), merged with the TCGA data and corrected to calculate the gene expression differences in cancers. Data from tumor cell line were acquired from the CCLE database (https://portals.broadinstitute.org/ccle/) and gene expression levels in these tumor tissues were explored according to tissue origin. Furthermore, the association between expression and cancer stage was investigated.

#### Prognosis correlation analysis

The overall survival (OS) and progression-free interval (PFI) data of TCGA cases were downloaded from the Xena dataset to further explore the association between gene expression and patient prognosis. Survival analysis (p < 0.05) for each cancer type was conducted using the Kaplan–Meier method, and survival analyzes were evaluated using the 'survival' and 'survminer' packages. Additionally, Cox analysis employed the "survival" and "forestplot" packages to evaluate the relationship between gene expression and prognosis.

#### Immune cell infiltration analysis

The CIBERSORT algorithm was taken to analyze the RNA-seq data of 33 cancer patients in different subpopulations to explore the relative proportion of immune cells, and to perform correlation analysis on gene expression and immune cell content. In addition, potential relationships between gene expression and immune modulators (chemokines, immunosuppressants, immune stimulators, and MHC molecules, etc.) were explored using TISIDB website.

#### Tumor mutation burden analysis

TMB is known as the total number of detected somatic genetic coding errors, base substitutions, insertions, or deletions per million bases. In this research, TMB was defined by dividing the non-synonymous mutation site by the total length of the protein coding region through calculating the variant frequency and variant number/exon length for each tumor sample. The MSI values for each TCGA case were derived from a previously published study^9^.

#### Drug sensitivity analysis

The Cellminer database is based on 60 cancer cell types listed by the National Cancer Institute Cancer Research Center (NCI). The NCI-60 cell line is presently the most generally used cancer cell sample group for anticancer drug analysis. This study acquired NCI-60 drug sensitivity data and RNA-seq expression data, and explored the association between genes and common antitumor drug sensitivity through correlation analysis, and p < 0.05 was considered significant.

#### GSVA enrichment analysis

Gene Set Variation Analysis (GSVA) is a nonparametric and unsupervised manner for evaluating gene set enrichment in the transcriptome. GSVA converts gene-level changes into pathway-level changes through comprehensively scoring the gene set of interest, and then judges the biological function of the sample. In this study, the gene set will be downloaded from the Molecular signatures database (version 7.0), and the GSVA algorithm will be used to score each gene set comprehensively to evaluate the potential biological function changes of different samples.

#### GSEA enrichment analysis

Gene Set Enrichment Analysis (GSEA) analysis uses a preordinated gene set to sort the genes based on the degree of differential expression in the two kinds of samples, and then checks whether these gene set is enriched at the top or bottom of the sorting table. In this study, the "clusterprofiler" and "enrichplot" packages were used for GSEA analysis, and the possible underlying mechanism of the difference in prognosis of different patients in 33 tumors was explored by comparing the differences in signaling pathways between the high and low gene expression groups.

#### Nomogram model construction

Nomogram is built on multi-factor regression analysis, according to gene expression and clinical symptoms, and then uses scaled line segments to draw on the same plane according to the certain ratio, so as to express the relationship between variables in these model mutual relationship. By constructing a multi-factor regression model, according to the contribution degree of each influencing factor in the model to the outcome variable (the size of the regression coefficient), assign a score to each value level of each influencing factor, and then add the scores to get the total score to calculate the predicted value.

#### WGCNA analysis

Through constructing a weighted gene co-expression network, find co-expressed gene modules, and explore the association between gene network and phenotype, as well as the core genes in the network. The WGCNA-R package was used to construct the co-expression network of all genes in the glioma data set, and the top 5000 genes with variance were filtered by this algorithm for further analysis. The weighted adjacency matrix is converted into a topological overlap matrix (TOM) to analyze the network connectivity, and the hierarchical clustering method is taken to construct the clustering tree structure of the TOM matrix. In addition, different branches of the clustering tree represent different gene modules, and different colors represent different modules. According to the weighted correlation coefficient of genes, genes are classified according to their expression patterns, genes with similar patterns are grouped into one module, and all genes are divided into multiple modules by gene expression patterns.

#### Gene function validation

In this research, siRNA targeting SLC35C1 was used to knock down the expression of SLC35C1 mRNA. Construction of Pklv2-SLC35C1 plasmid for the establishment of glioma cell lines stably overexpressing SLC35C1. The CCK-8 assay was used to detect cell proliferation. EdU assay is used to detect the amount of cell proliferation. Plate clone formation assay is used to detect cell growth and proliferation ability as well as colony formation. Transwell as well as wound healing/scratch assays were used to evaluate cell invasion and migration. Quantitative real-time PCR was taken to detect the expression of SLC35C1 in glioma tissues. Detailed materials and methods are elucidated in Supplementary Materials and Methods.

#### Statistical analysis

All statistical analyzes were performed using the R language (version 4.0). Hazard ratios (HRs) and 95% confidence intervals were calculated using univariate survival analysis. Kaplan–Meier analysis was used to study the survival of patients according to the high or low level of gene expression. All statistical tests were two-sided, and p < 0.05 was considered statistically significant.

### Results

#### Pan-cancer expression analysis of SLC35C1 gene

**Figure 1:**
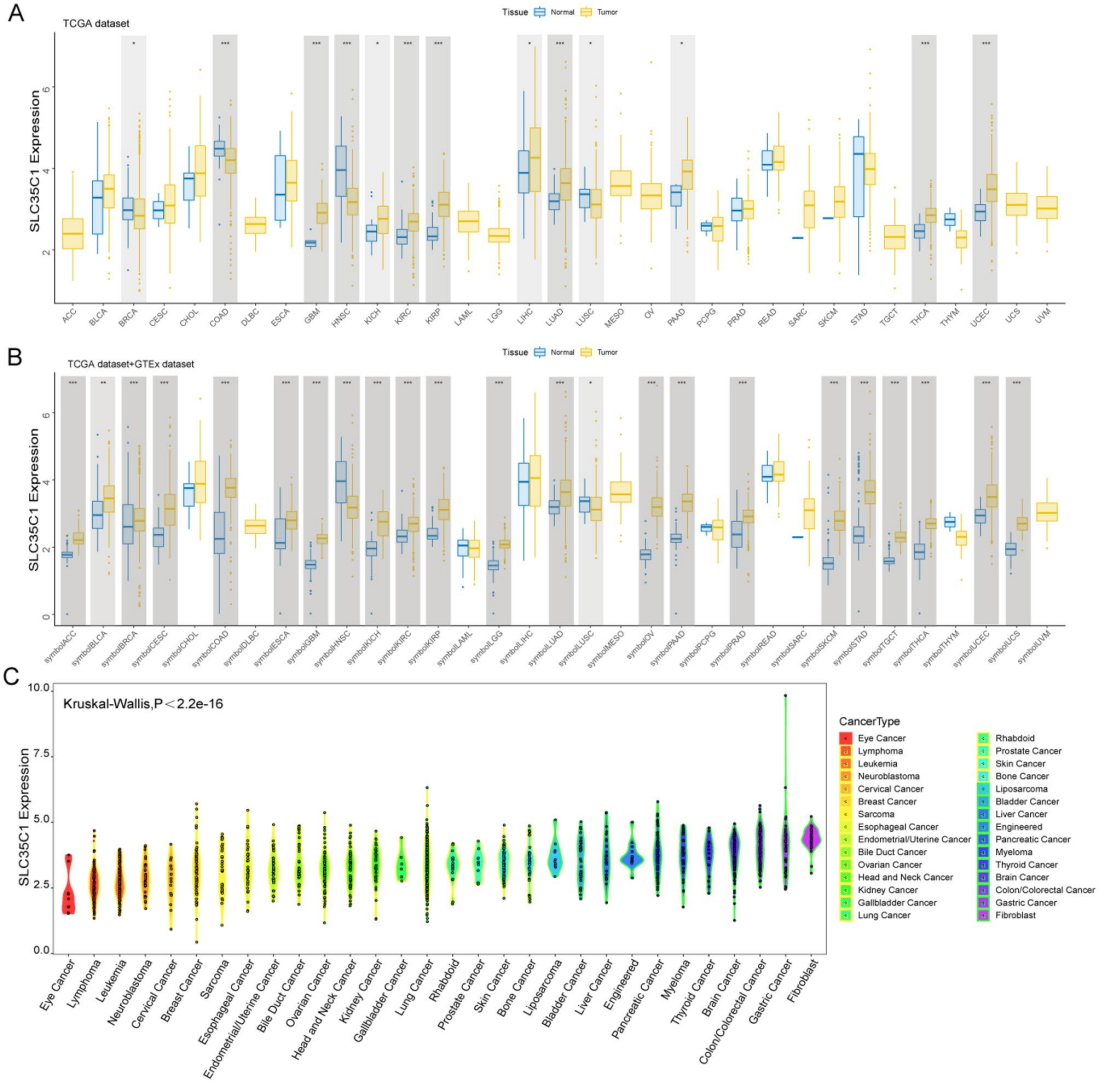
The SLC35C1 expression level in adrenocortical carcinoma (ACC), bladder urothelial carcinoma (BLCA), breast invasive carcinoma (BRCA), Cervical squamous cell carcinoma and endocervical adenocarcinoma (CESC), cholangiocarcinoma (CHOL), colon adenocarcinoma (COAD), Lymphoid Neoplasm Diffuse Large B-cell Lymphoma (DLBC), esophageal carcinoma (ESCA), Glioblastoma multiforme (GBM), head and neck squamous cell carcinoma (HNSC), kidney chromophobe (KICH), kidney renal clear cell carcinoma (KIRC), kidney renal papillary cell carcinoma (KIRP), Acute Myeloid Leukemia (LAML), Brain Lower Grade Glioma (LGG), liver hepatocellular carcinoma (LIHC), lung adenocarcinoma (LUAD), lung squamous cell carcinoma (LUSC), mesothelioma (MESO), Ovarian serous cystadenocarcinoma (OV), pancreatic adenocarcinoma (PAAD), prostate adenocarcinoma (PRAD), rectum adenocarcinoma (READ), Sarcoma (SARC), skin cutaneous melanoma (SKCM), stomach adenocarcinoma (STAD), testicular germ cell tumors (TGCT), Thyroid carcinoma (THCA), Thymoma (THYM), Uterine Corpus Endometrial Carcinoma (UCEC), Uterine Carcinosarcoma (UCS) and uveal melanoma (UVM). (**A**) The mRNA level of SLC35C1 in TCGA. The color refers to the tumor (yellow) or normal (blue), respectively. (**B**) The WASF2 expression level in 33 types from the GTEx database and TCGA database. (**C**) SLC35C1 expression in 30 tumor cells from CCLE database. *P < 0.05; **P < 0.01; ***P < 0.001.

**Figure 2:**
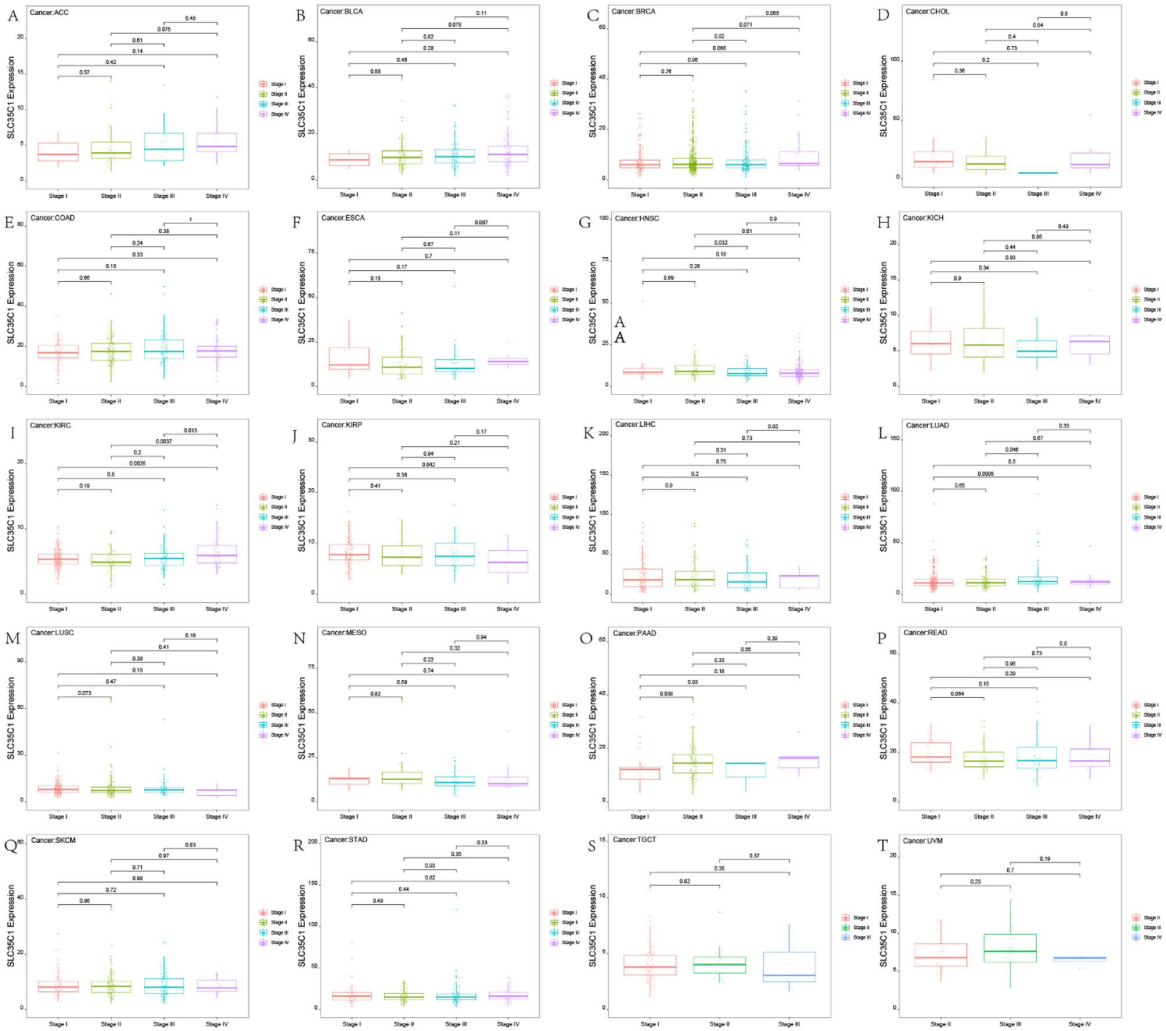
The box plot shows the association of SLC35C1 expression with pathological stages in (**A**) ACC, (**B**) BLCA, (**C**) BRCA, (**D**) CHOL, (**E**) COAD, (**F**) ESCA, (**G**) HNSC, (**H**) KICH, (**I**) KIRC, (**J**) KIRP, (**K**) LIHC, (**L**) LUAD, (**M**) LUSC, (**N**) MESO, (**O**) PAAD, (**P**) READ, (**Q**) SKCM, (**R**) STAD, (**S**) TGCT, and (**T**) UVM. Kruskal–Wallis test was used to assess the significance of differences between groups, followed by pair wise comparisons using Dunn’s multiple comparisons test used to evaluate differences among groups.

**Figure 3:**
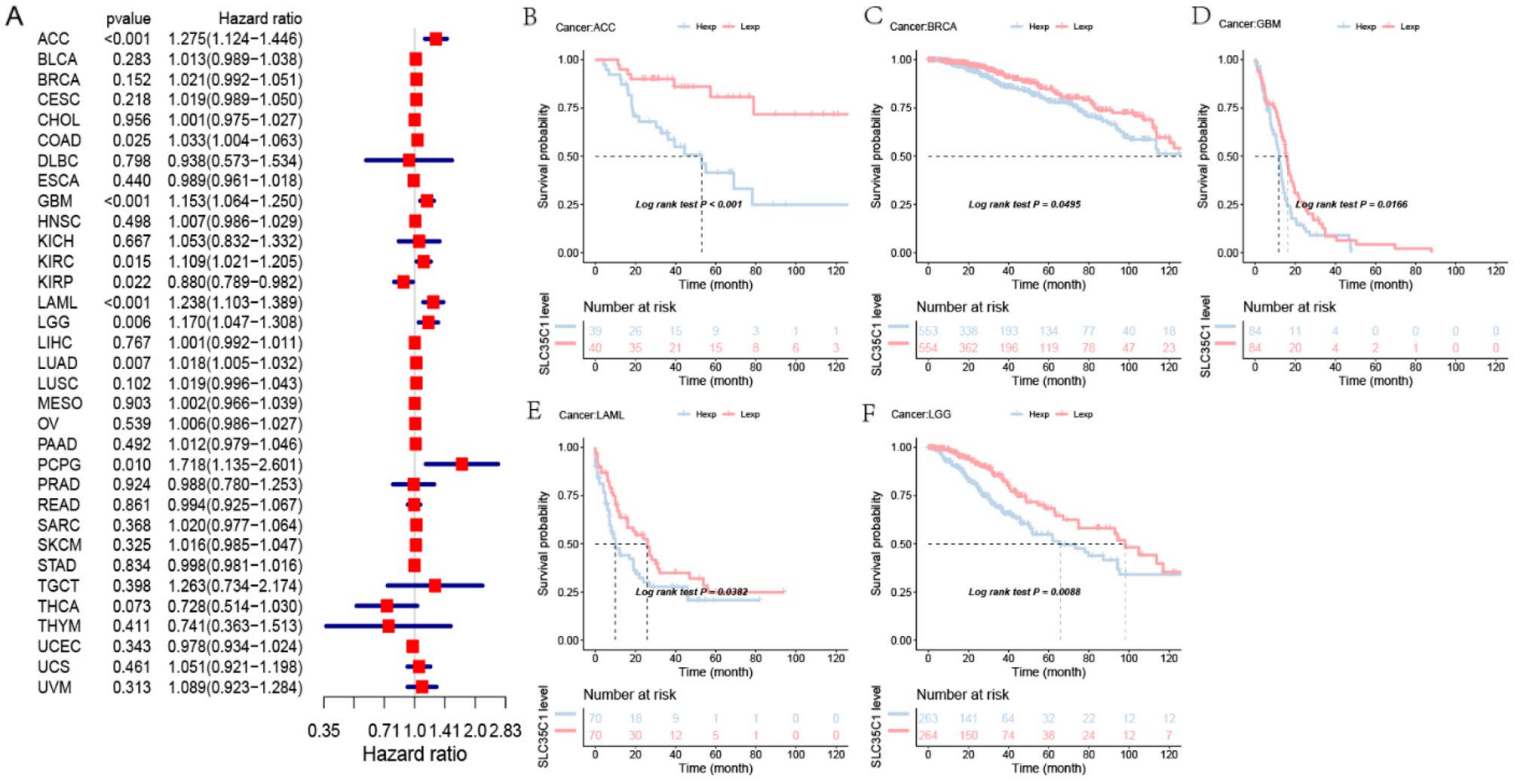
Association of SLC35C1 expression with patient overall survival (OS) in pan- cancer. (**A**) Forest plot of HR for the relationship between SLC35C1 expression and patient OS. (**B**–**F**) Kaplan–Meier analyses show the association between SLC35C1 expression and OS. Statistical significance was assessed using the log-rank test.

**Figure 4:**
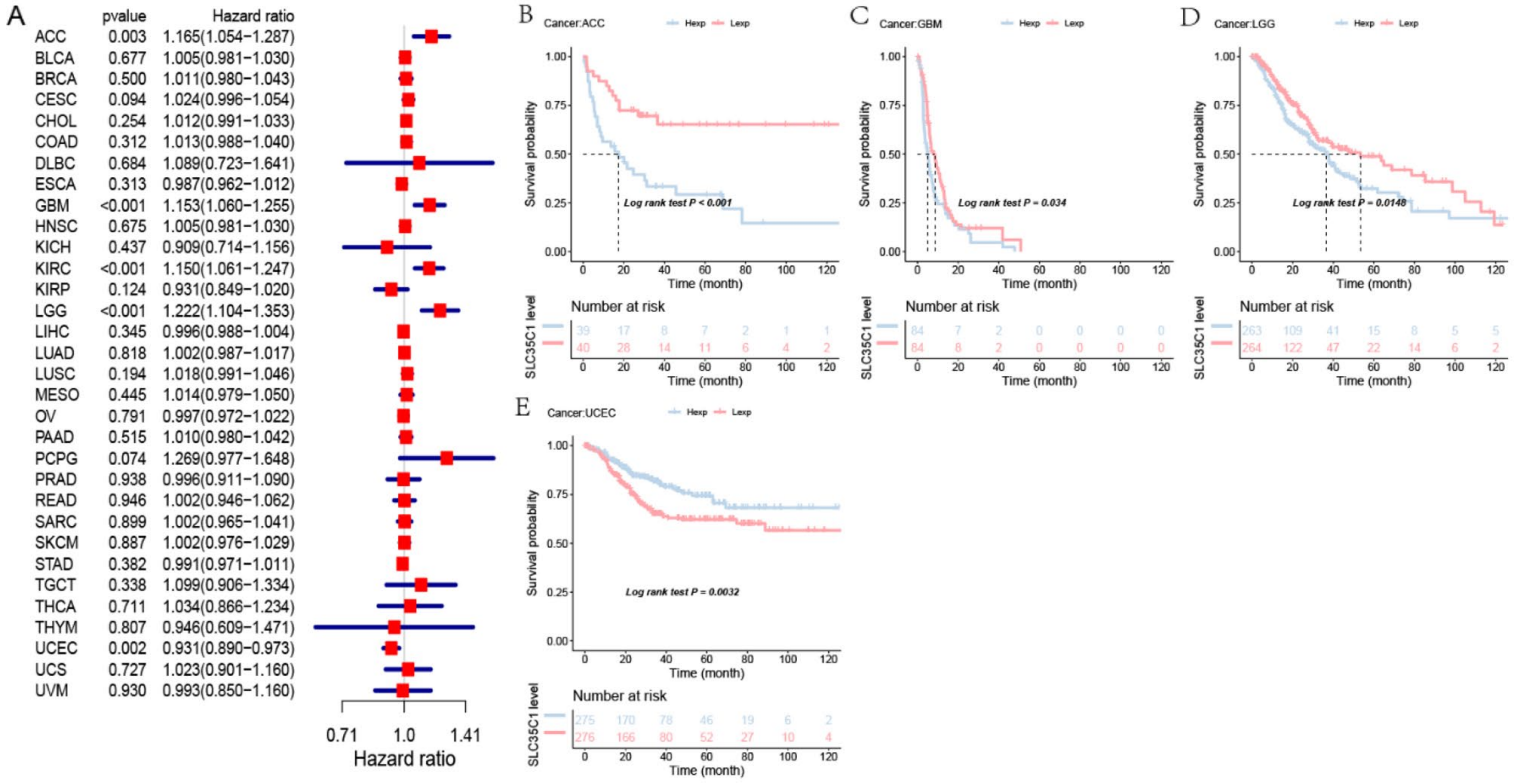
Association of SLC35C1 expression with patient progression-free interval (PFI) in pan- cancer. (**A**) Forest plot of HR for the relationship between SLC35C1 expression and patient PFI. (**B**–**E**) Kaplan–Meier analyses show the association between SLC35C1 expression and PFI. Statistical significance was assessed using the log-rank test.

The expression of SLC35C1 in 33 cancers in humans was evaluated using TCGA and GTEx datasets. The results showed that the gene was highly expressed in 9 kinds of tumors, such as GBM, KICH, KIRC, KIRP, LIHC, LUAD, PAAD, THCA, and UCEC (Fig. 1A). In primarily normal tissues, the expression level of SLC35C1 was lower than that in tumors. Because of the small number of normal tissue cases in TCGA database, the normal tissue data from the GTEx database and the tumor tissue data from TCGA database were combined to explored the differences of SLC35C1 expression in 33 cancers. These results displayed that SLC35C1 was abnormally expressed in 23 of these tumors. Specifically, SLC35C1 expression was higher in 21 cancer (ACC, BLCA, BRCA, CESC, COAD, ESCA, GBM, KICH, KIRC, KIRP, LGG, LUAD, OV, PAAD, PRAD, SKCM, STAD, TGCT, THCA, UCEC and UCS) and lower in 2 cancers (HNSC, LUSC) compared with the normal tissues (Fig. 1B). The expression of SLC35C1 in different tumor cell lines in the CCLE expression profile is displayed in the figure (Fig. 1C). Also, SLC35C1 is associated with various tumor stages, such as HNSC, KIRC, KIRP, LUAD, PAAD (Fig. 2). We estimated the relationship between SLC35C1 expression and the prognosis of cancer patients, with survival indicators including OS and PFI. The results showed that the expression of SLC35C1 was closely related to the OS of 9 cancer patients, including ACC, COAD, GBM, KIRC, KIRP, LAML, LGG, LUAD, and PCPG tumors (Fig. 3A); In addition, the results of KM-plot analysis showed that SLC35C1 It was associated with poor OS of 5 types of cancer, including: ACC, BRCA, GBM, LAML, and LGG (Fig. 3B–F). The expression of SLC35C1 is closely related to the PFI of 5 kinds of cancer patients, such as ACC, GBM, KIRC, LGG, and UCEC tumors (Fig. 4A). The KM-plot results suggest that SLC35C1 is related to the poor PFI of 3 kinds of cancers, including ACC, GBM, LGG (Fig. 4B–E).

#### Pan-cancer expression and immune infiltration

**Figure 5:**
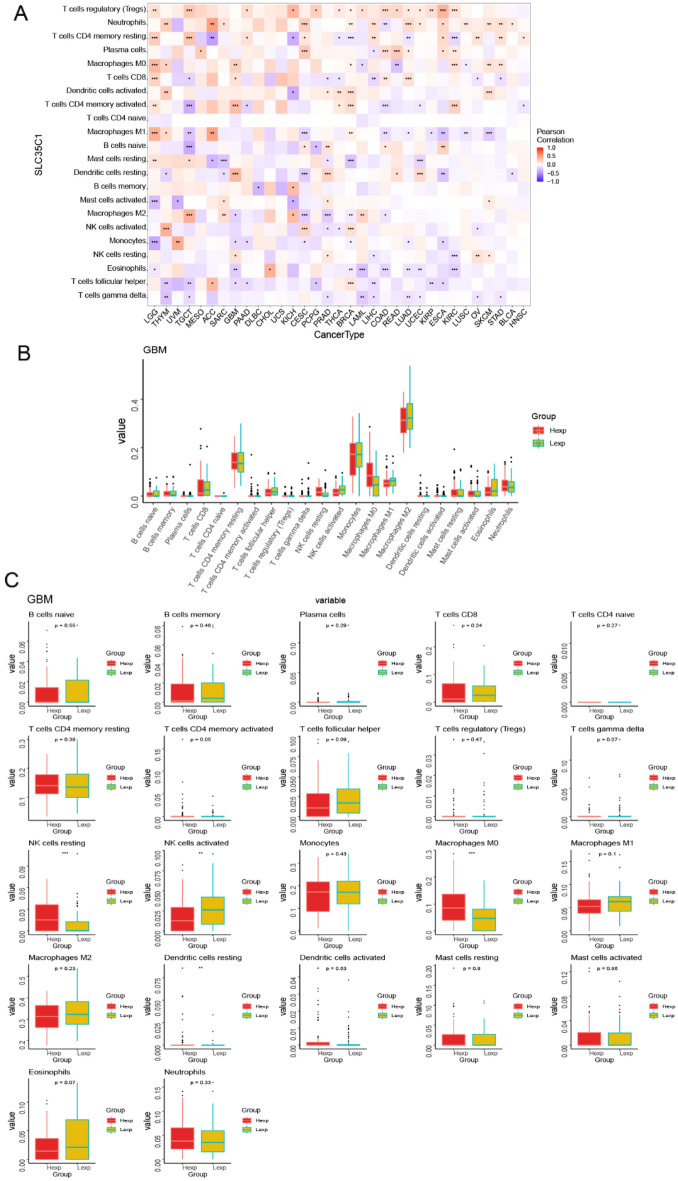
SLC35C1 expression is correlated with cancer immunity. (**A**) Correlation between the expression of SLC35C1 and infiltration by 22 types of immune cells in pan-cancer analysis. Red denotes a correlation coefficient > 0, whereas blue denotes a correlation coefficient < 0. (**B**, **C**) The statistical chart after using the CIBERSORT method shows the proportion difference of immune cell between SLC35C1 high and low expression groups in ovarian cancer. Red represents the high SLC35C1 expression group, yellow represents the low SLC35C1 expression group. *P < 0.05; **P < 0.01; ***P < 0.001. ns, no significant.

**Figure 6:**
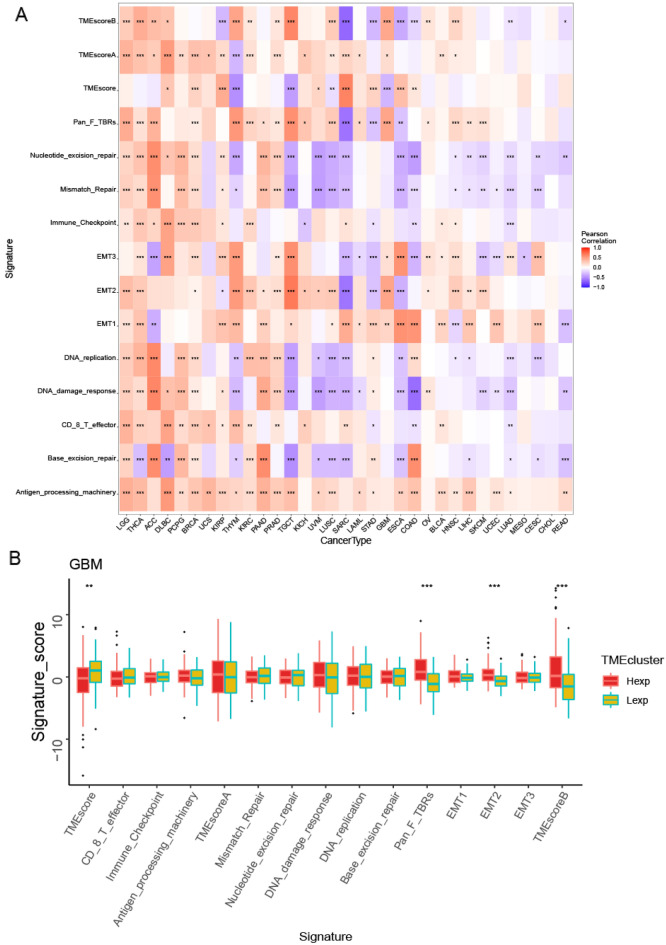
SLC35C1 expression is correlated with the TME. (**A**) Correlation between the expression of SLC35C1 and 15 TME processes. Red denotes a correlation coefficient > 0, whereas blue denotes a correlation coefficient < 0. (**B**) The statistical chart after using the CIBERSORT method shows the proportion difference of TME between SLC35C1 high and low expression groups in glioma. Red represents the high SLC35C1 expression group, yellow represents the low SLC35C1 expression group. *P < 0.05; **P < 0.01; ***P < 0.001. ns, no significant.

The tumor microenvironment is primarily including tumor-associated fibroblasts, immune cells, extracellular matrix, various growth factors and so on. The tumor microenvironment originally affects the diagnosis and survival outcome of tumors and clinical sensitivity to treatment. These results showed that the expression of SLC35C1 was closely associated with immune infiltration, among which 15 cancers were significantly related to T cells regulatory (Tregs) cells, 14 cancers were significantly related to Neutrophils cells, and 15 cancers were significantly related to T cells CD4 memory resting cells (Fig. 5A). We conducted further immune infiltration analysis on GBM, and the results presented that NK cells resting, NK cells activated, macrophages M0 and dendritic cells resting were significantly related to GBM (Fig. 5B,C). Our results displayed that the expression of SLC35C1 was closely related to TME (Fig. 6A). We further analyzed the tumor microenvironment of glioma, and the results showed that TMEscore, Pan_F_TBRs, EMT2, and TMEscoreB scores were all significantly correlated with glioma (Fig. 6B).

#### The relationship between SLC35C1 expression and key regulatory genes

**Figure 7:**
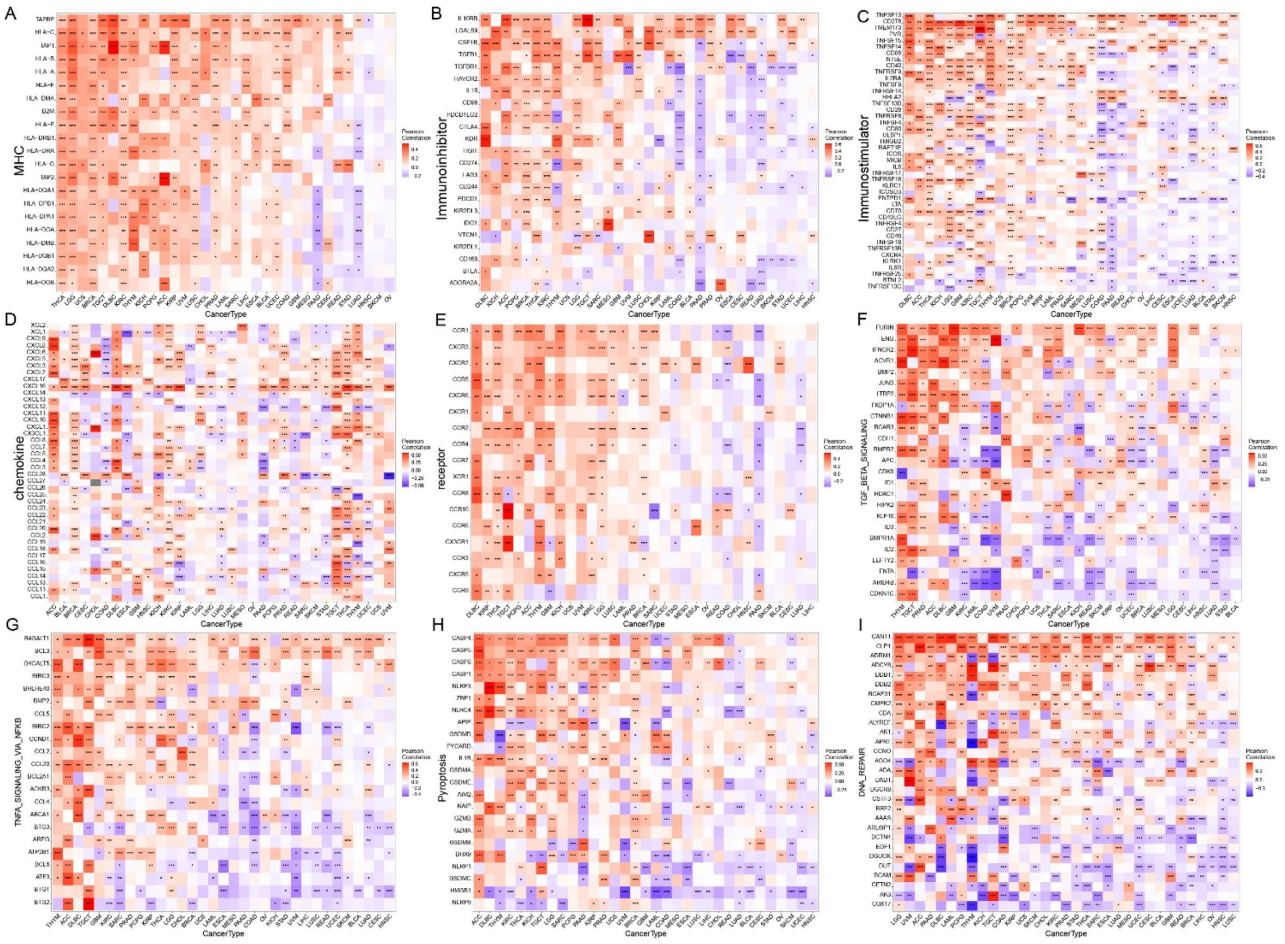
SLC35C1 expression is correlated with Immune-related genes. (**A**) The correlation between SLC35C1 and MHC gene. (**B**) The correlation between SLC35C1 and Immunoinhibitor gene. (**C**) The correlation between SLC35C1 and Immunostimulator gene. (**D**) The correlation between SLC35C1 and Chemokine gene. (**E**) The correlation between SLC35C1 and Receptor gene. (F) The correlation between SLC35C1 and TGF_BETA_SIGNALING gene. (**G**) The correlation between SLC35C1 and TNFA_SIGNALING_VIA_NFKB gene. (**H**) The correlation between SLC35C1 and Pyroptosis gene. (**I**) The correlation between SLC35C1 and DNA_REPAIR gene. *P < 0.05; **P < 0.01; ***P < 0.001.

In this study, gene co-expression study was further performed to analyze the relationship between SLC35C1 expression and 33 tumor immunity-associated genes. Genes analyzed included MHC, immunosuppressors, immunostimulants, chemokines, and chemokine receptor proteins. The results showed that almost all immune-associated genes were significantly related to SLC35C1 (Fig. 7A–E). In addition, SLC35C1 is significantly correlated with common tumor-related regulatory genes such as TGF BETA SIGNALING, TNFA SIGNALING, pyroptosis, DNA repair, autophagy genes, ferroptosis-related genes, immune checkpoint (Fig. 7F–I and Supplement Fig. 1A–C).

#### Pan-cancer expression and TMB and MSI

**Figure 8:**
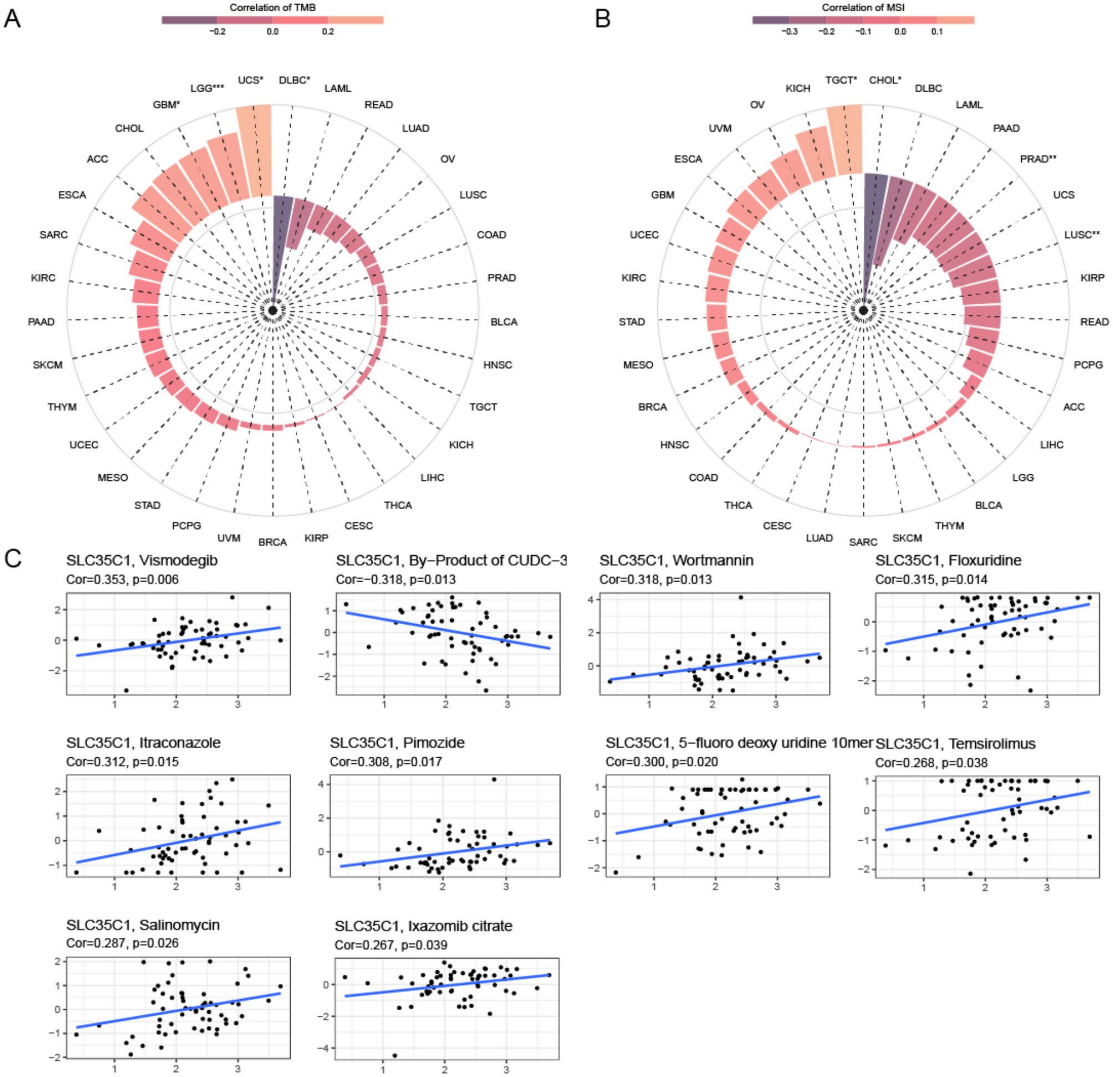
SLC35C1 expression is correlated with TMB, MSI and drug sensitivity. (**A**) Correlation analysis between SLC35C1 expression in pan-cancer and TMB described using Spearman’s rank correlation coefficient. (**B**) Correlation analysis between SLC35C1 expression in pan-cancer and MSI described using Spearman’s rank correlation coefficient. (**C**) Analysis of drug sensitivity associated with SLC35C1. The positive correlation means that the gene’s high expression is resistant to the drug, while the negative is the opposite. *P < 0.05; **P < 0.01; ***P < 0.001.

TMB and MSI are new biomarkers associated with immunotherapy response. This study analyzed the relationship between the expression of SLC35C1 and TMB and MSI. The results presented that the expression level of SLC35C1 was significantly correlated with TMB tumors, including significant differences in GBM, LGG, UCS, and DLBC (Fig. 8A). In MSI, the gene SLC35C1 was significantly different in TGCT, CHOL, PRAD, and LUSC (Fig. 8B).

#### Pan-cancer expression and drug sensitivity

Early tumors can be cured by surgery combined with chemotherapy. We used the Cellminer database to study the correlation between the SLC35C1 gene and anti-tumor drugs, and found that the high expression of the gene SLC35C1 was predicted to be related to resistance to multiple anti-tumor drugs (Fig. 8C). Among them, SLC35C1 was positively associated with Ixazomib citrate, Vismodegib, Wortmannin and other drugs, and negatively correlated with By-Product of CUDC-305.

#### Association of SLC35C1 expression with GSVA and GSEA in ovarian cancer

**Figure 9:**
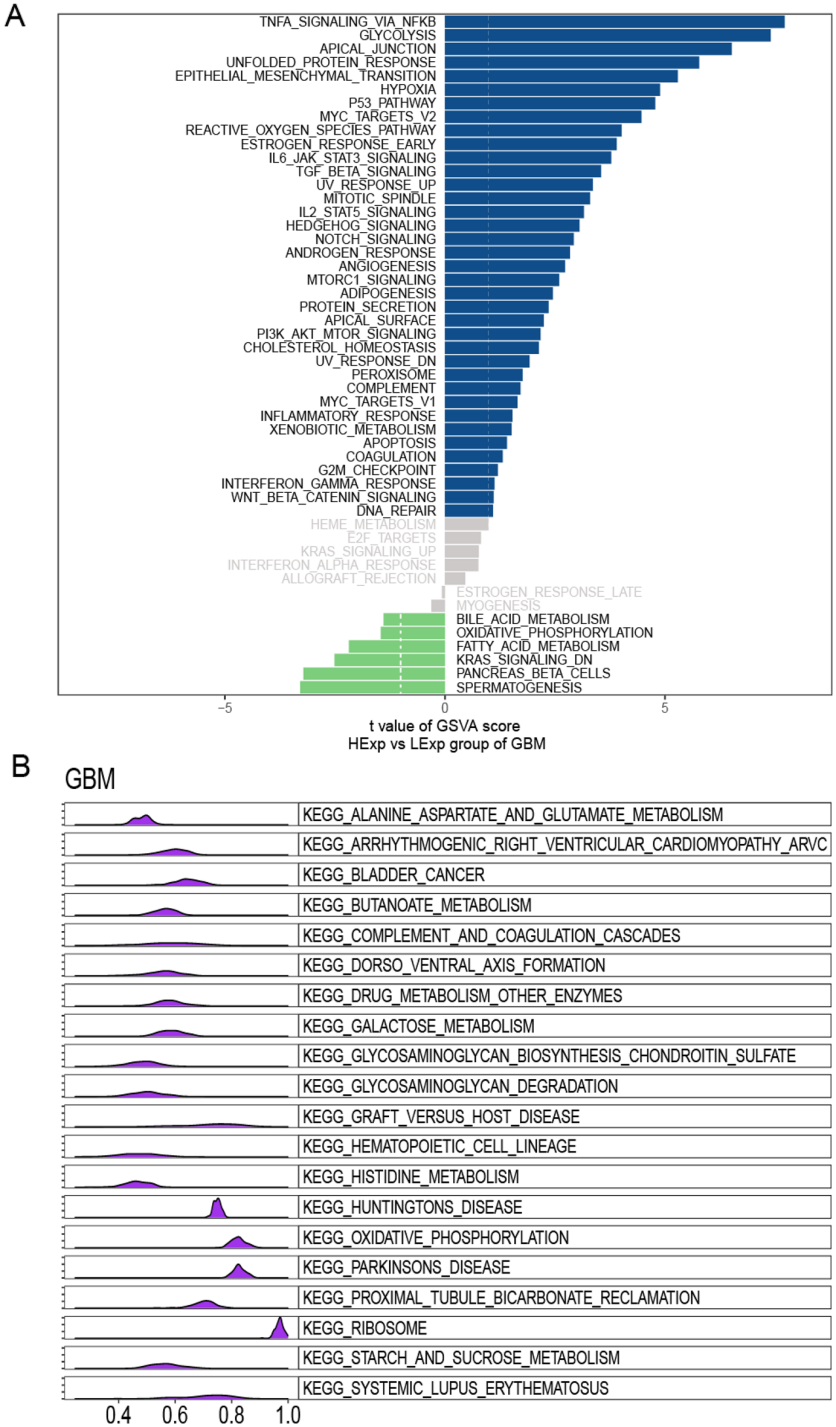
Function and pathway enrichment analysis of glioma. (**A**) Correlation analysis results of GSVA and SLC35C1 in glioma. (**B**) KEGG results of SLC35C1 GSEA in glioma.

In order to deeply study the molecular mechanism of SLC35C1 gene in pan-cancer, we firstly scored all tumors cases with GSVA, and then divided the samples into two groups with high and low expression based on the median of gene expression for comparison between the two groups. The results displayed that in glioma, the high expression of SLC35C1 was mainly concentrated in TNFA_SIGNALING_VIA_NFKB, GLYCOLYSIS, APICAL_JUNCTION and other signaling pathways (Fig. 9A). The GSEA analysis of SLC35C1 and glioma tissue is shown in the figure (Fig. 9B).

#### SLC35C1 risk and independent prognosis analysis

**Figure 10:**
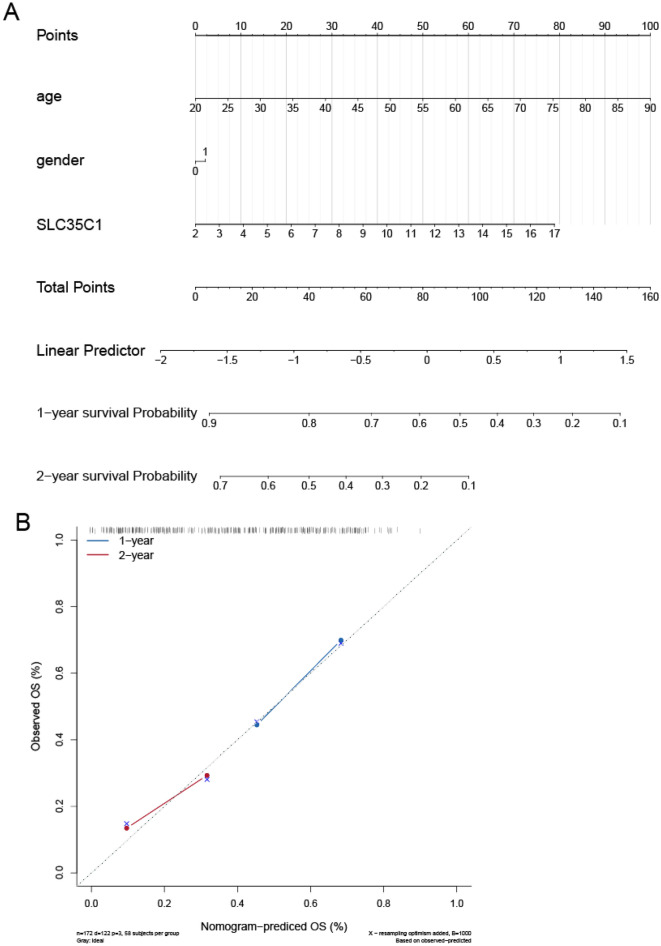
Establishment and validation of the prognostic nomogram. (**A**) Nomogram based on the SLC35C1 signature and clinical information for prediction of the 1- and 2-year OS in patients with glioma in the TCGA dataset. (**B**) The calibration curves is used to verify the consistency of predicted and actual 1-,2-year outcomes.

According to the findings of the Cox regression analyses, this study further constructed a nomogram based on the age, grade, and the expression of SLC35C1, to create a quantitative method for clinicians to predict the probability of 1‐ and 2‐year OS in glioma patients (Fig. 10A). To evaluate the score, each prognostic parameter was projected to the value of the small ruler (points), with a higher number of total points presenting a worse prognosis for the cases. Also, the calibration curve for the 1‐ and 2‐year OS was plotted at the same time, and the nomogram showed a good performance (Fig. 10B).

#### WGCNA analysis

**Figure 11:**
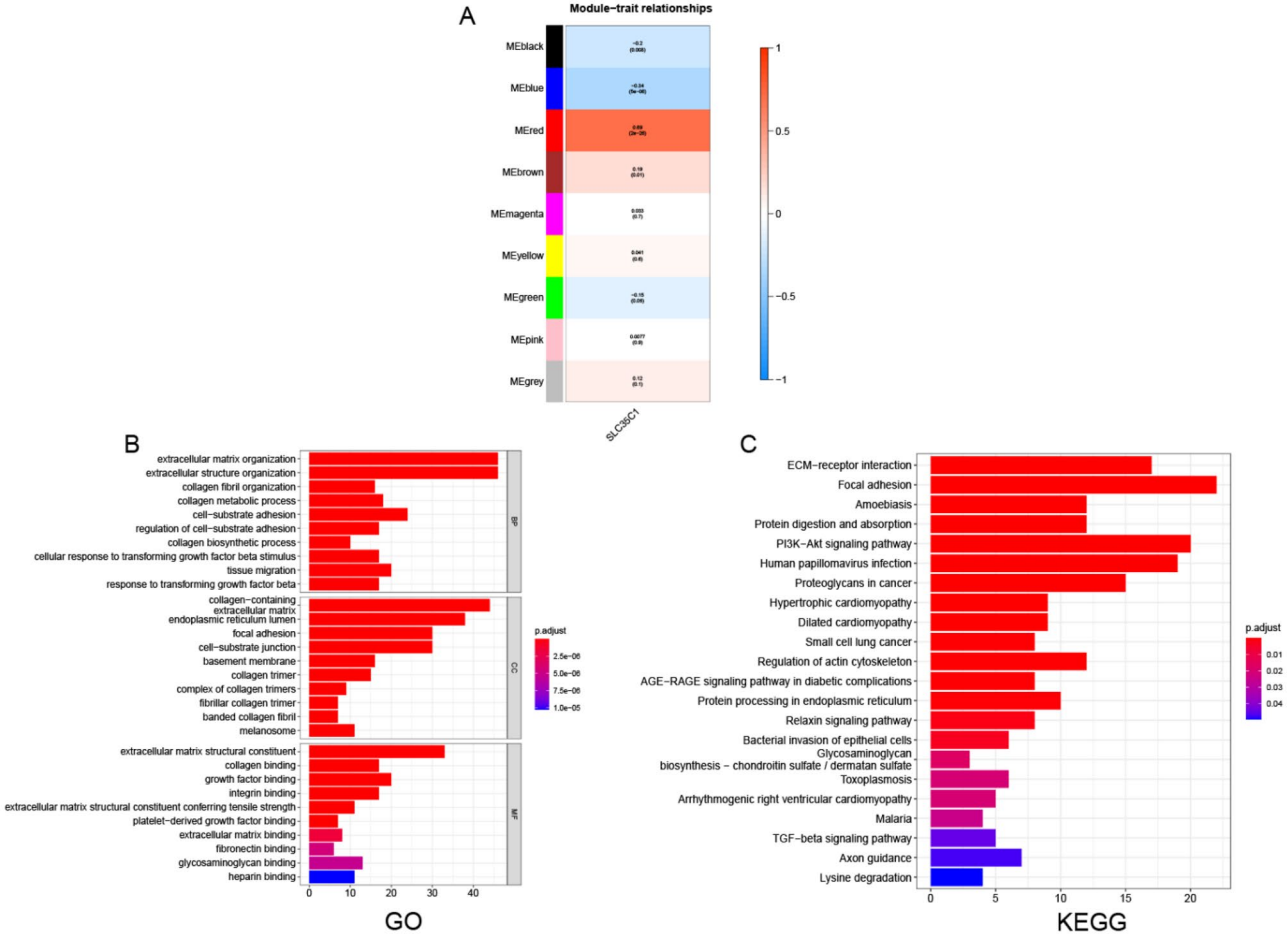
Association of SLC35C1 expression with WGCNA in glioma. (**A**) Module trait relationship (p-value) for detected modules (y-axis) in relation with traits (x-axis) for glioma. The relationships were colored based on the correlation between the identified module and traits. The color scale on the right demonstrate module-trait relationship from − 1 (blue) to one (red), where blue represents strong negative correlation and red represents a strong positive correlation. (**B**) Gene ontology (GO) and corresponding P-values are shown. (**C**) Kyoto Encyclopedia of Genes and Genomes (KEGG) and corresponding P-values are shown.

We further constructed the WGCNA network according to glioma data to explore the regulatory network related to SLC35C1 in glioma. The soft threshold β is evaluated by the function "sft$powerEstimate", and then the gene modules are detected based on the tom matrix. A total of 9 gene modules were used in this analysis, namely black (158), blue (766), brown (488), green (1438), gray (1405), magenta (69), pink (79), red (175), yellow (422). We further analyzed the relationship between modules and traits, and found that the red module had the highest correlation with SLC35C1 (cor = 0.69, p = 2e − 26) (Fig. 11A). We further used the red module genes for pathway analysis. Also, GO results showed that the genes were mainly enriched in extracellular matrix organization, extracellular structure organization, collagen fibril organization and other pathways (Fig. 11B). KEGG results showed that genes were mainly enriched in pathways such as Focal adhesion, ECM-receptor interaction, and PI3K-Akt signaling pathway (Fig. 11C).

#### SLC35C1 knockdown and overexpression

**Figure 12:**
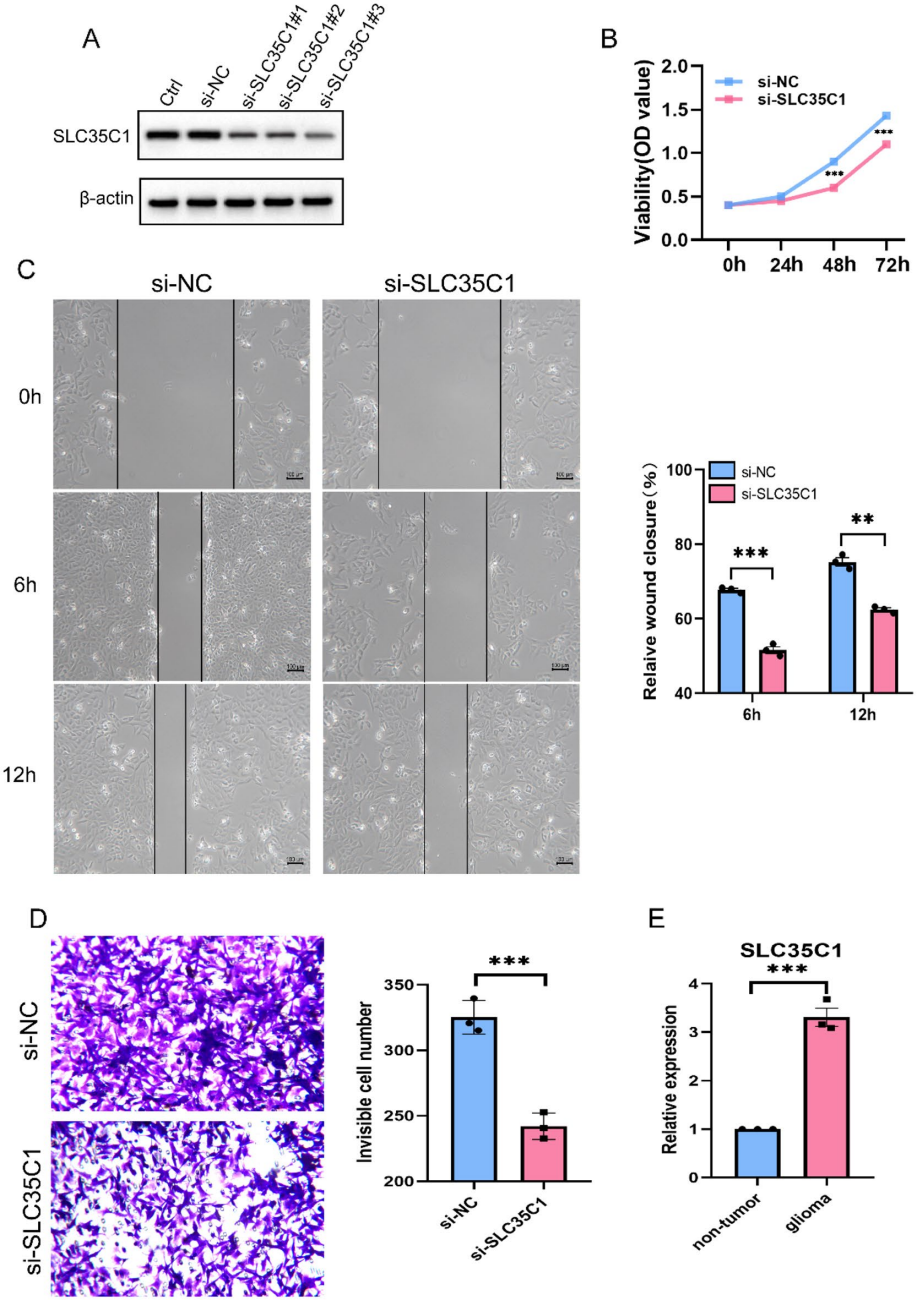
Knockdown of SLC35C1 inhibits the proliferation, migration and invasion capacities of glioma cells in vitro. (**A**) Western blot analysis of transfection efficiency of the siRNA. original blots/gels are presented in Supplementary Fig. 6 (**B**) Cell proliferation was detected by using the CCK8 proliferation reagent. (**C**) Effect of SLC35C1 knockdown in cell migration was determined by wound healing assay and the percentage of scratch-width closure measured by quantifying the images the scratch assay at 0, 6 and 12 h after incubation. (**D**) Invasiveness of glioma cells analyzed by transwell invasion assay (magnification, × 100) and bar graph showing quantitative results of the transwell assay. (**E**) Validations for long non-coding RNAs expressions. Detection of significant differences in the expression levels of SLC35C1-related lncRNA in non-tumor and glioma by qPCR. *P < 0.05; **P < 0.01; ***P < 0.001.

**Figure 13:**
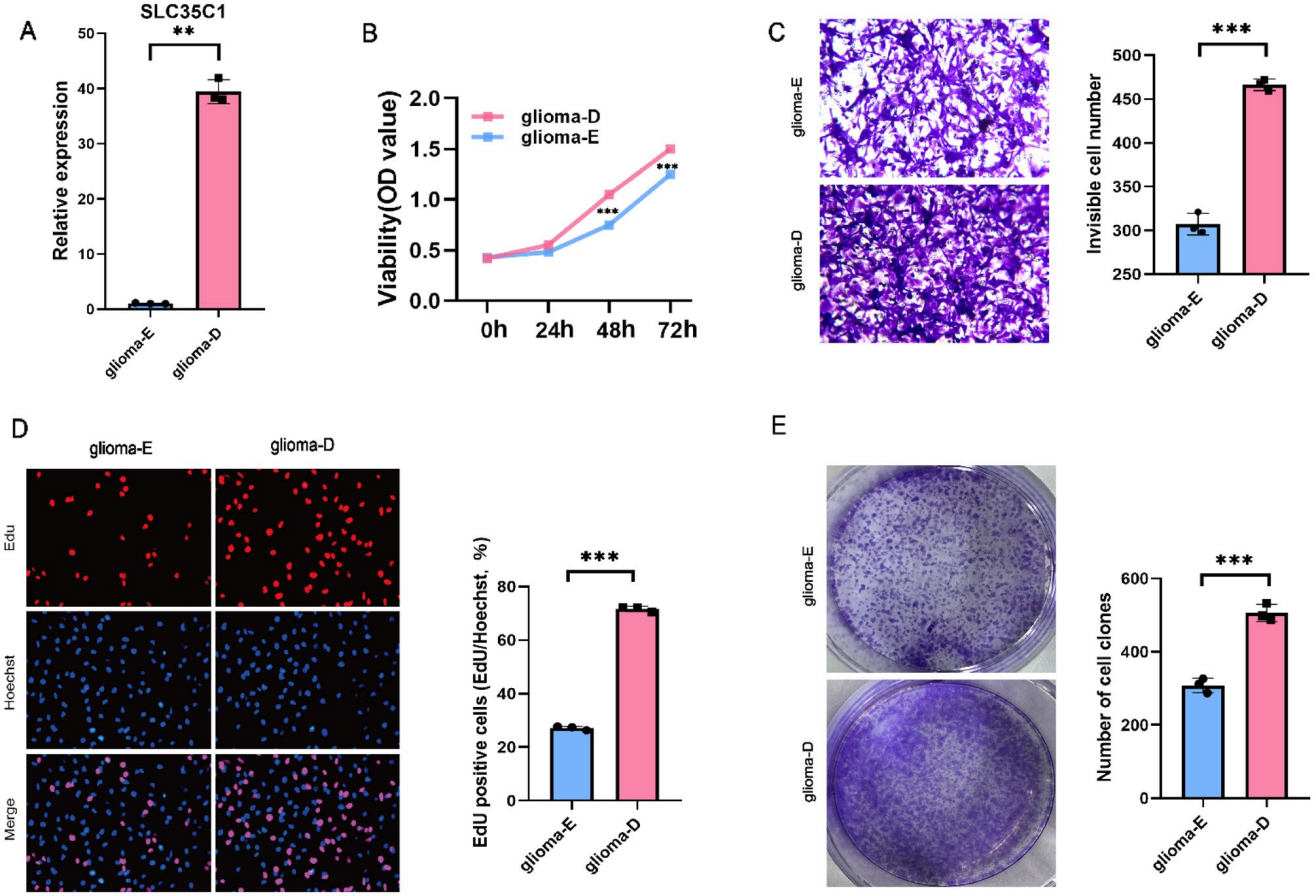
Overexpression of SLC35C1 promotes proliferation, migration, invasion and colony formation of glioma cells in vitro. (**A**) PKlv2-SLC35C1 plasmid (glioma-D) and Empty vector (glioma-E) were stably transfected into glioma cells, respectively, and the expression of SLC35C1 was detected by qRT-PCR. (**B**) Cell proliferation was detected by using the CCK8 proliferation reagent. (**C**) Invasiveness of glioma cells analyzed by transwell invasion assay (magnification, × 100) and bar graph showing quantitative results of the transwell assay. (**D**) The picture shows the PKlv2-SLC35C1 plasmid (glioma-D) group and the empty vector (glioma-E) group EdU-labeled cells, Hoechst-stained cells, and Edu merged with Hoechst; Bar graphs analyzed the rate of Edu-positive cells in the two groups. (**E**) Plate clone formation assay detects cell proliferation capacity and colony formation. *P < 0.05; **P < 0.01; ***P < 0.001.

To further investigate the role of SLC35C1 in the migration and invasion of glioma cells, we performed knockdown and overexpression of SLC35C1. specific siRNA was used to silence SLC35C1 expression (Fig. 12A). The effect of silencing SLC35C1 on glioma cell proliferation was examined by CCK8 assay, which presented that SLC35C1 knockdown significantly inhibited cell proliferation (Fig. 12B). Also, we found that upon SLC35C1 knockdown, cells showed significantly slower wound area closure than control cells and significantly reduced invasion potential compared to their respective control cells (Fig. 12C). Subsequent Quantitative real-time PCR further confirmed that the expression of SLC35C1 was significantly increased in glioma tissues (Fig. 12D). In addition, we constructed a glioma cell line stably overexpressing SLC35C1 (Fig. 13A). The effect of overexpression of SLC35C1 on glioma cell proliferation was examined by CCK8 assay, and the results showed that SLC35C1 overexpression significantly promoted cell proliferation (Fig. 13B). Transwell assay shows significantly enhanced cell invasion potential after SLC35C1 overexpression (Fig. 13C). The results of EdU assay and Plate clone formation assay showed that overexpression of SLC35C1 significantly promoted the proliferation and colony formation of glioma cells (Fig. 13D,E). These results showed that SLC35C1 is important in the migration and invasion of gliomas.

### Discussion

SLC35C1 is a typical solute carrier (SLC) gene, and its encoded guanosine 5′-diphosphate (GDP)-fucose transporter 1 channel can support fucosylation of glycans. The research results show that this gene mutation will cause leukocyte adhesion defect, which may affect the biosynthesis of selectin ligand and cause related diseases^10^. Some scholars found that fucosylation belongs to the post-translational modification mode of some oncogenes^11^. Moriwak found that SLC35C1 was highly expressed in hepatocellular carcinoma, and the corresponding fucosylation level was significantly higher than that of normal hepatocytes^12^. Deng's found that the expression level of SLC35B1 was reduced in various colon cancers, and its silencing could accelerate the progression of cancer based on the Wnt signal pathway^5^. However, in other human cancers, the role of this gene is unclear. Under this context, this paper used R software and multiple databases (including TCGA, GTEx, CCLE, Xena) for statistical analysis, and discussed the role of SLC35C1 in pancancer, and provides reference for research in this field. The study found that SLC35C1 is highly expressed in various human cancer tissues and cells, and its expression level is closely related to the prognosis of patients, which can be regarded as a biomarker of pan-cancer. Its expression level is correlated with TMB, MSI, TME, immunomodulators and drug reactions. Finally, the results of in vitro experiments showed that the silencing and overexpression of SLC35C1 had completely opposite effects on the physiological activity of glioma cells.

Our research showed that SLC35C1 gene is highly expressed in 9 tumors and lowly expressed in 4 tumors. In this study, we found that the expression of SLC35C1 decreased in colorectal cancer, which is different from previous studies, suggesting that SLC35C1 may be a potential prognostic marker. The reason for this discrepancy was because of the differences in tumor samples, as former research have included more metastatic cancers. And recently, Deng et al. found that SLC35C1 is a negative regulator of the classical Wnt pathway in colon cancer, and the deletion of SLC35C1 promotes colon cancer progression by activating the Wnt signaling pathway, a finding that further confirms our view^5^. We found that the expression of SLC35C1 was significantly different between normal brain tissue and glioma, suggesting that SLC35C1 has a crucial role in the occurrence and development of glioma, which will provide new possibilities for the treatment of glioma^6^ As we have learned, critical analysis of tumor survival prognostic factors plays an important role in the formulation of clinical treatment decisions. In addition, we found that the expression of SLC35C1 was closely related to the staging and grading of tumors, including HNSC, KIRC, KIRP, LUAD, PAAD. Therefore, we analyzed the relationship between SLC35C1 expression and survival. We found that poor prognosis and short overall survival in ACC, GBM, LAML, and LGG were usually associated with high expression of SLC35C1, but SLC35C1 appears to be a protective factor for KIRP. Therefore, the study of the differential expression of SLC35C1 in a variety of tumors and its mechanism of action is of great clinical significance.

At present, many scholars have studied tumor metabolism and tumor microenvironment, and TME has become a hot topic in the field of tumor mechanism research^13–15^. In the clinical treatment of tumor, the immune microenvironment composed of infiltrating lymphocytes and other immune cells is of great significance^16–19^. Some scholars have found that immune cells can play two regulatory roles on tumor cells. Specifically, under normal physiological conditions, immune cells can play an anti infection role, and can also kill cancer cells^20–24^. At the same time, cancer cells can escape the immune clearance system based on a variety of ways^25,26^. Some scholars have found that macrophages in TME can polarize into M2 macrophages under certain specific conditions, and this immune microenvironment formed by cell transformation can promote the growth of cancer cells^27^. Related studies also found that in the anti-tumor immune environment, CD3+, CD4+ and CD8 + cells and CD4+/CD8+ will have a direct impact on immune function^3^. Neutrophils can promote the immune escape of tumor cells to some extent, which is mainly based on the promotion of tumor angiogenesis and tumor cell activity^28,29^. At present, there have been many studies related to this, but the specific relationship between immune cell infiltration and SLC35C1 still needs to be further investigated. Based on the results of the above analysis, we found that there was a high correlation between SLC35C1 expression and the degree of immune infiltration. In addition, we analyzed the relationship between the expression of SLC53C1 and three immune modes (immune stimulators, immune inhibitors, and MHC molecules). In ACC and DLBC, the expression of SLC35C1 was positively correlated with TAP1. A previous study reported that TAP1 promoted chemoresistance by enhancing the transport of MEKi out of PDAC cells, leading to reduced intracellular MEKi concentration and attenuated inhibition of KRAS signaling pathways^30^. Therefore, SLC35C1 can be a potential target to provide a new strategy for the treatment of PDAC by interfering with TAP1. Meanwhile, SLC35C1 and CD276 showed a significant co-expression relationship in a variety of tumors. And current evidence suggests that CD276 is involved in regulating the recruitment of tumor-associated macrophages and is an upstream regulator of PAI-1, with a strong correlation between its overexpression in tumors and poor prognosis^31–34^. Finally, we also found that SLC35C1 is also correlated with the expression of many tumor regulatory genes such as TGFβ, SIGNALING, DNA repair, and autophagy genes, and regulates these processes accordingly. For example, SLC35C1 and CXCL16 showed a clear co-expression relationship in the majority of tumors. The current studies have also fully demonstrated that the CXCL16 → CXCR6 axis plays an important role in the proliferation and migration of tumor cells, intercellular communication in tumor niche, angiogenesis, and recruitment and differentiation of various cells in tumor niche^35^. The above results also suggest that SLC35C1 is critical for the development of cancer pathology and significantly affects patient prognosis, correlating with the pathological process of cancer.

TMB is an extremely important tumor predictive biomarker^36^, which plays an indispensable role in predicting the clinical benefit rate of tumor patients after receiving immune checkpoint inhibitors^37,38^. Previous studies have shown that non-small cell lung cancer and colon cancer patients with high TMB have a higher clinical benefit rate after immunotherapy^39,40^. MSI is also an important clinical tumor marker that can guide more precise individualized targeted immunotherapy^38,41^. Our research shows that SL35C1 expression correlated with TMB in 4 tumors and with MSI in 4 tumors. This indicates that abnormal expression of SLC35C1 can affect the response of patients to ICI by affecting the TMB and MSI of tumor. This provides effective guidance for precise immunotherapy of tumors.

Surgery combined with chemotherapy is still the preferred treatment for most early tumors. So we further analyzed the Cellminer database and that the occurrence and development of glioma was closely related to the elevated expression of SLC35C1^42^. It was positively correlated with the sensitivity of four drugs (Ixazomib, cirtrate, Vismodegib, Wortmannin) and negatively correlated with the sensitivity of By-Product of CUDC-305. The results showed that SLC35C1 could be used as a biological predictor to evaluate drug resistance and drug sensitivity of tumor cells, thus providing new ideas for subsequent clinical research.

Then we further explored the mechanism of glioma development by GSVA and GSEA analysis. Enrichment analysis shows that SLC35C1 may act mainly through signaling pathways such as TNFA_SIGNALING_VIA_NFKB, GLYCOLYSIS, and APICAL_JUNCTION thus affecting glioma development and progression. The WGCNA results show that these genes are mainly enriched in extracellular matrix tissues, extracellular structural tissues, collagen fiber tissues and other pathways. These results also suggest that SLC35C1 is involved in a variety of biological processes that promote cancer development. This further confirms our previous study. Deng et al. found that reduced levels of SLC35C1 may increase nuclear translocation of β-linked proteins, and that β-linked proteins activate signaling cascades that ultimately lead to tumorigenesis^5^. Numerous studies have shown that overexpression of SLC35C1 significantly promotes tumor cell migration and invasion, and that tumor cell migration and invasion can be inhibited by downregulating SLC35C1. Therefore, we performed in vitro experiments to validate the results and showed that knockdown and overexpression of SLC35C1 have completely opposite effects on glioma cell genesis and development.

Although we performed a comprehensive pan-cancer analysis of SLC35C1, its limitations require further discussion. First, the data used in this study were from publicly available sources, and although we refined our in vitro experiments, other public databases are needed to validate our conclusions. Second, SLC35C1 was highly expressed in pan-cancer and correlated with clinical outcomes; however, its potential mechanism with rockweed glycosylation in tumors remains to be further investigated. Finally, this study found that SLC35C1 is closely related to tumor immunity, but the study of its specific molecular mechanism is still insufficient.

In conclusion, our systematic pan-cancer analysis revealed the biological characteristics of SLC35C1 in cells and tissues and found that SLC35C1 is associated with the risk and prognosis of a variety of tumors. Our existing research results show that SLC35C1 is an independent prognostic factor for a variety of tumors, and its expression level shows different prognostic results in different tumors. Therefore, the specific role of SLC35C1 in various tumors needs to be further explored. Based on the current findings, we believe that SLC35C1 expression is closely related to immune infiltration and a potential marker of TME. Furthermore, SLC35C1 expression was associated with TMB, MSI, and antitumor drug sensitivity. Finally, we established a risk factor pattern based on SLC35C1 expression could predict OS of glioma. Also, fluorescence quantitative PCR further confirmed the high expression of SLC35C1 in glioma patients. These findings will further clarify the role of SLC35C1 in tumorigenesis and development, providing guidance for precise immunotherapy.

## Supplementary Information

Supplementary Information.

## Data Availability

The data used to support the findings of this study are included in the article.
