# Supplementary material for: Systematic pan-cancer analysis identifies SLC35C1 as an immunological and prognostic biomarker
Source: Sci Rep. 2023 Apr 1;13:5331. doi: 10.1038/s41598-023-32375-0 (PMC10067962; doi:10.1038/s41598-023-32375-0)
Supplement: Supplementary file 1 — Supplementary Information. [file 41598_2023_32375_MOESM1_ESM.docx]

**Methods**

Cell culture and quantitative real‑time PCR

The glioma cell lines (U251) were purchased from Procell (Wuhan, China), and maintained in Dulbecco’s Modified Eagle’s Medium (Thermo Fisher Scientific, Waltham, MA, USA) with 10% fetal bovine serum (Gibco, Gran Island, NY, USA) and 1% penicillin/streptomycin (Beyotime, Shanghai, china) at 5% CO2 and 37 °C. The medium was changed every 3 days. Total RNA was isolated using TRIzol reagent, quantitative real-time fluorescence quantitative PCR(qRT-PCR) was performed by using primers (Beijing Genomics institution) and premix (SYBR Master Mix). Gene expression levels were calculated using the 2-ΔΔCt method. Actin was used as an internal control. Primers used in this study are listed in the table below.


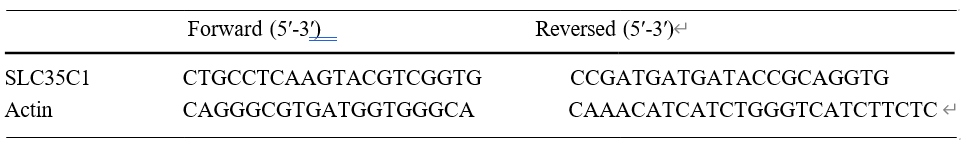


Transfection with small interfering (si) RNA

siRNAs targeting SLC35C1 and control siRNA were synthesized and purified by Heyuan Biotechnology Co., Ltd. (Shanghai, China). Transfection was performed using Lipofectamine® 3000 Transfection reagent (Invitrogen; Thermo Fisher Scientific, Inc.) following the manufacturer's protocols. Final concentration of siRNAs was 100 nM and cells were harvested 72 h following transfection.

Western blot analysis

Western blot analysis was performed following standard procedures. Total protein was extracted from U251 cells using RIPA lysis buffer containing a protease inhibitors (BiYunTian, Shanghai, China), and protein concentrations were detected using the BCA protein assay kit (Thermo Fisher Scientific, Inc.). Total protein (30 µg/lane) was loaded into each well by SDS-PAGE using a 10% gel and then transferred to a polyvinylidene fluoride (PVDF) membrane. The membrane was then blocked with 5% nonfat dry milk for 1 hour at room temperature. Following overnight incubation at 4°C with rabbit mAb to SLC35C1 (1:1,000; cat.no. 3659, RRID: AB_2216981). Subsequently, the membrane was incubated with the corresponding anti-rabbit secondary antibody (1:5,000; cat. no. ab6721; Abcam) for 2 hours at room temperature and detected by chemiluminescence. β-actin(1:1,000; cat. no. ab8226; Abcam) was used as a loading control.

Construction of Pklv2-slc35c1 plasmid

PCR amplification was performed using F:5` -tgtcgtgggaagcttggcgcgccatgaacagggcgcctgaa-3` and R:5`- taatccagaggttgagcggccgctcacaccctgatagcactcttctca-3` as primers, using mouse gDNA as a template. The Vazyme Phanta Max Super-Fidelity DNA Polymerase amplification system is as follows: 2 × Phanta Max Buffer 25ul,dNTP Mix (10 mM each) 1ul,Phanta Max Super-Fidelity DNA Polymerase 1ul, Stencil 10ng, primers F (10uM) and R (10uM) 2ul each, dd H2O to make up to 50ul. Reaction conditions: 95oC pre-deformation for 3min, 95oC denaturation for 15s, 60oC annealing for 15s, 72oC extension for 40s, complete 35 cycles, 72oC for 5min. The PCR products were recovered by gel, and the amplified fragments were inserted into the AscI and Notl polyclonal sites of PKLV2 using the vazyme ClonExpress II One Step Cloning Kit to obtain the plasmid PKLV2-slc35c1 transformed into E.coli DH5α receptor cells, after which single colonies were picked and the plasmids were extracted for enzymatic digestion After that, single colonies were picked and plasmids were extracted for enzymatic digestion and plasmid sequencing.

Establishment of glioma cells overexpressing slc35c1 cell line

Pklv2-slc35c1 and the helper plasmids PMD2G and PsPAX2 were cotransfected into 293T cells with LipofectamineTM2000 for Pklv2- slc35c1 lentivirus packaging. Afterwards, virus suspensions were collected and infected with glioma cell lines to establish glioma cell lines stably overexpressing slc35c1.

Cell proliferation assay

Cell proliferation was performed using Cell Counting Kit-8 (CCK8) (KeyGEN, Nanjing, China). Firstly, cultured cells were seeded into 96-well plates (1000 per well). Then, 10 μl CCK-8 solutions were added into each well at 24, 48, and 72 hours. Cells were incubated at 37℃ for 2h. The absorbance of each sample was measured with a microplate reader at a wavelength of 450nm.

Wound healing assay

A total of 3×105 cells per well were seeded in 6-well plates, then transfected with SLC53C1 siRNA following overnight incubation. When cells reached 90% confluence, the cell monolayer was scratched with a sterile pipette tip and cells were cultured in DMEM for 12 h. Photographs were taken at 0, 6 and 12 h along the scrape line using a light microscope. Migration images were captured by an inverted microscope and the relative percentage of wound closure was determined by comparison with control cells.

Invasion assay

Invasion analysis was conducted by Transwell inserts with polycarbonate membranes (8.0 μm pore size) with Matrixgel (BD). U251 cells (4×104 cells/well) were seeded into the upper chamber and culture medium containing 20% FBS was added in the lower chamber. After 24h of incubation, non-migrated cells in the upper chamber were removed with cotton swabs. The migrated cells were fixed with 4% paraformaldehyde , stained with crystal violet and counted.

EdU assay

U251 cells were divided into 2 groups, including control (glioma-E) and SLC35C1 overexpression (glioma-D) groups. The cells of each group were inoculated into the cell chambers, and EdU reagent was added after 16 h. The cells were continued to be cultured for 4 h. The cells were removed and washed twice with PBS or 0.9% NaCl for 3 min each time. The cells were then slowly suspended and added dropwise to the slides to distribute the cells as evenly as possible, and then 0.5 ml of Hoechst 33258 staining solution was added dropwise and stained for 5 min. Hoechst mainly stained the nuclei of the cells to indicate the total number of cells in the field of view, and EdU mainly stained the mitotic cells to indicate the cells in the proliferation stage in the field of view.

Plate clone formation assay

The cells of each group were digested with 0.25% trypsin to prepare a single cell suspension, adjust the cell density, inoculate into 6-well plates, each group of cells inoculated with 3 wells, 300/well, gently shake the 6-well plates in the cross direction to make the cells evenly dispersed. The cells were incubated at 37℃, 5% CO2 incubator, and incubated for about 10 d. When the clones were visible to the naked eye, the culture medium was discarded, and the cells were carefully rinsed twice with PBS and fixed with 10 ml of methanol for 15 min. The fixative was discarded, and the appropriate amount of stain was added for 30 min, and then the stain was slowly washed off with running water, dried in air at room temperature, and counted under ordinary light microscope.

Supplementary figure legends

**
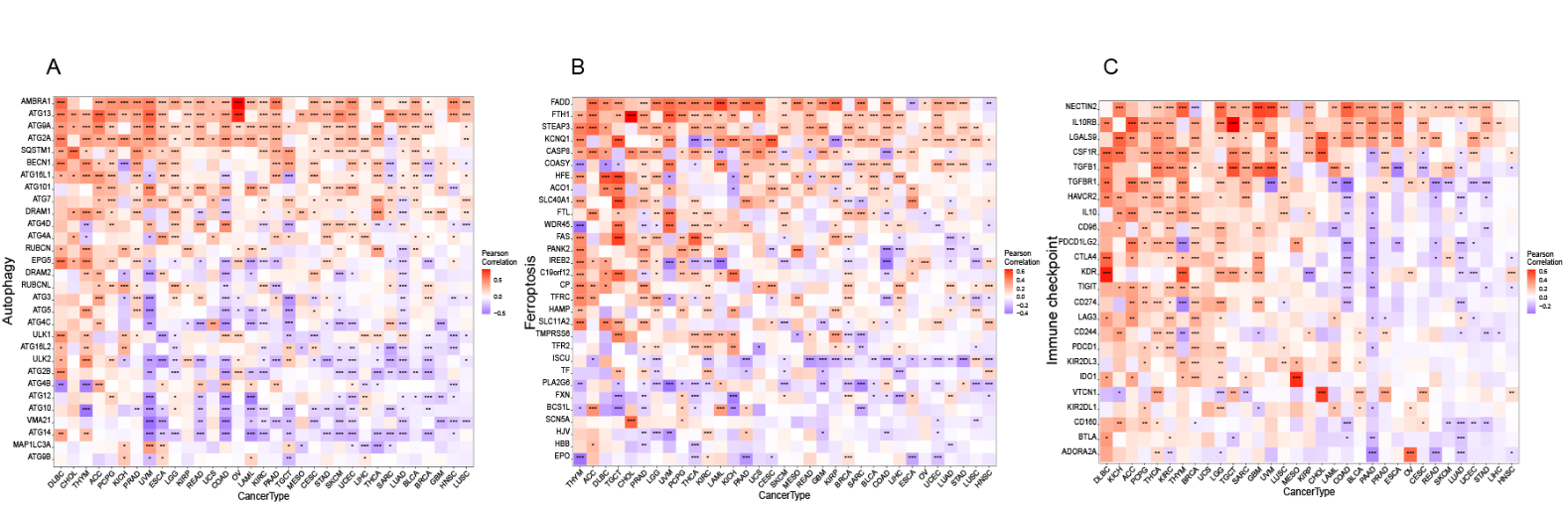
**

Supplement Figure1 : SLC35C1 expression is correlated with Immune-related genes. (A) The correlation between SLC35C1 and Autophagy gene. (B) The correlation between SLC35C1 and Ferroptosis gene. (C) The correlation between Immune checkpoint gene. *P < 0.05; **P < 0.01; ***P < 0.001.

**
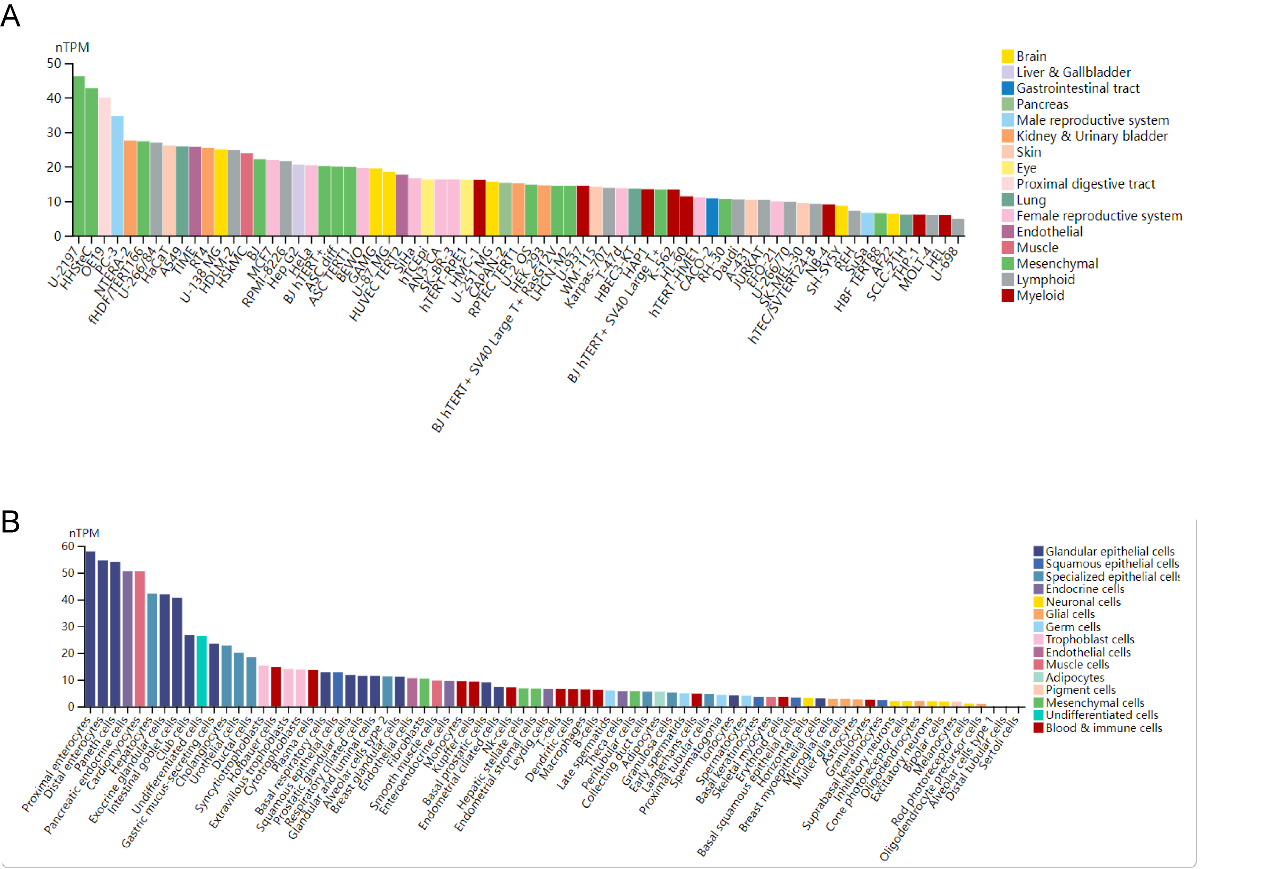
**

Supplement Figure2: (A)The expression data of SLC35C1 in different normal cells. (B) A summary of normalized single cell RNA (nTPM) from all single cell types. Color-coding is based on cell type groups, each consisting of cell types with functional features in common.


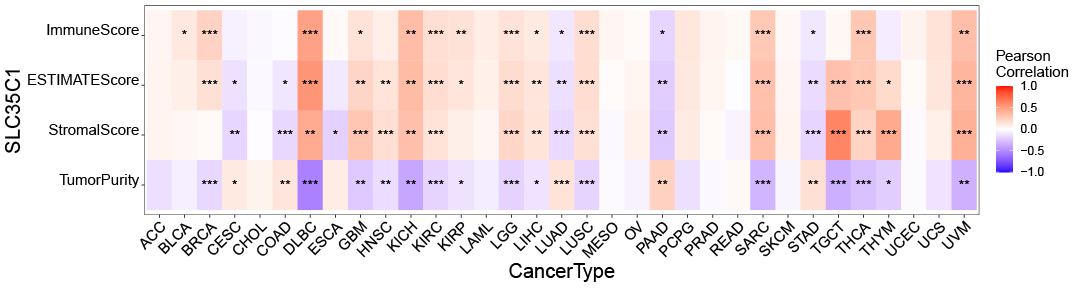


Supplement Figure3: Correlation analysis of SLC35C1C expression with ESTIMATE scores. Red indicates a correlation coefficient > 0, whereas blue indicates a correlation coefficient < 0.


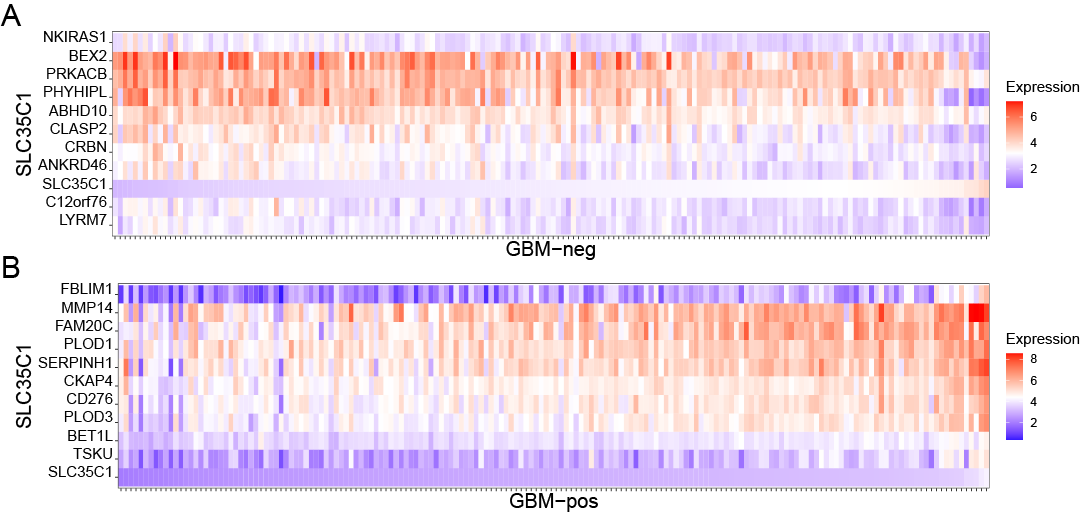


Supplement Figure4: Correlation analysis of SLC35C1 with multiple genes. Red indicates a correlation coefficient > 0, whereas blue indicates a correlation coefficient < 0.


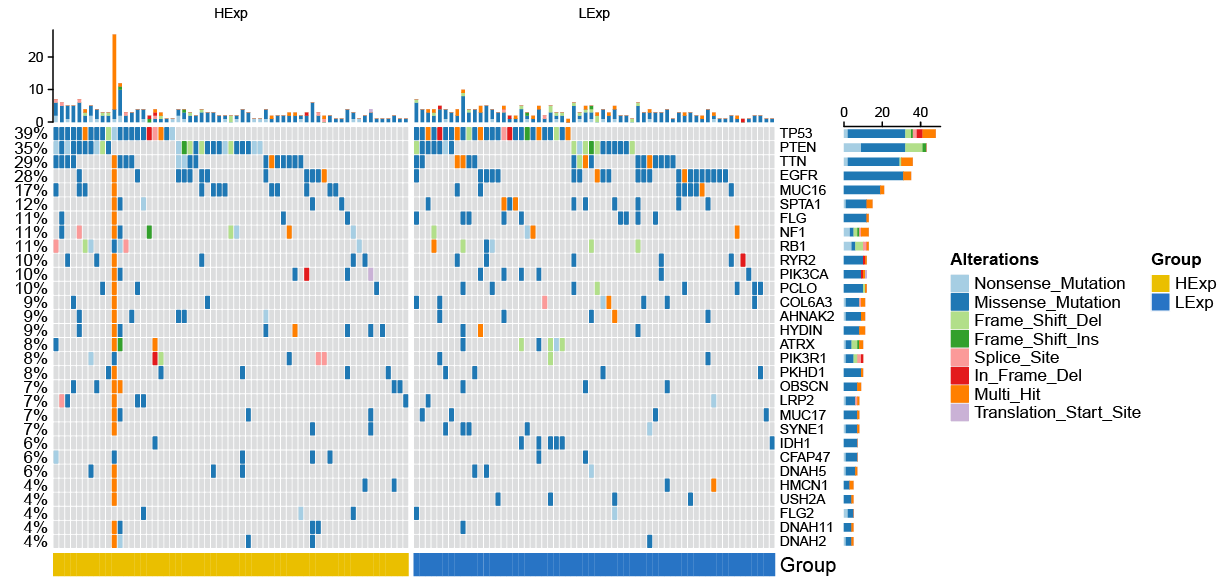


Supplement Figure5: The median value of SLC35C1 was used to group high and low expression, and the comparison of gene mutation differences in each tumor, the left side of each plot type represents the proportion of mutations and the right side represents the type of mutated genes. The left panel shows the gene mutation analysis in the high expression group, and the right panel shows the gene mutation analysis in the low expression group.


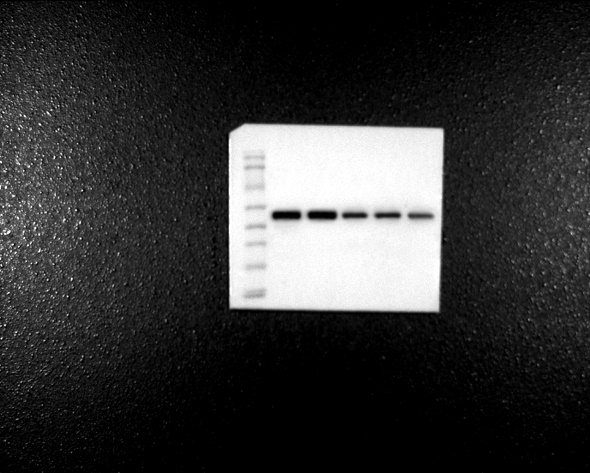

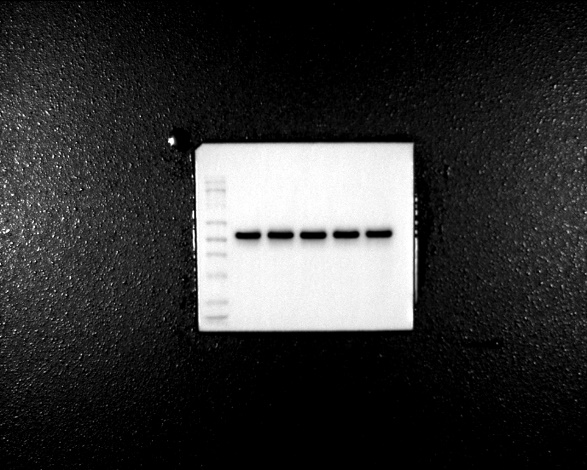


ACTIN si-SLC35C1

Supplement Figure6: Western blot analysis Uncropped graph.
